# Addressing equity, diversity, and inclusion in JBI qualitative systematic reviews: a methodological scoping review

**DOI:** 10.11124/JBIES-24-00025

**Published:** 2024-09-04

**Authors:** Catrin Evans, Zeinab M. Hassanein, Manpreet Bains, Clare Bennett, Merete Bjerrum, Alison Edgley, Deborah Edwards, Kylie Porritt, Susan Salmond

**Affiliations:** 1 The Nottingham Centre for Evidence-based Healthcare: A JBI Centre of Excellence, School of Health Sciences, University of Nottingham, Nottingham, UK; 2 Public Health and Community Medicine Department, Faculty of Medicine, Assiut University, Asyut, Egypt; 3 Nottingham Centre of Epidemiology and Public Health, School of Medicine, University of Nottingham, Nottingham, UK; 4 The Wales Centre For Evidence Based Care: A JBI Centre of Excellence, School of Healthcare Sciences, Cardiff University, Cardiff, Wales, UK; 5 Danish Centre of Systematic Reviews: A JBI Centre of Excellence, The Centre of Clinical Guidelines – Danish National Clearing House, Department of Clinical Medicine, Aalborg University, Aalborg, Denmark; 6 JBI, Faculty of Health and Medicine Sciences, The University of Adelaide, Adelaide, SA, Australia; 7 The Northeast Institute for Evidence Synthesis and Translation: A JBI Centre of Excellence, School of Nursing, Rutgers, The State University of New Jersey, Newark, NJ, USA

**Keywords:** diversity, equity, inclusion, qualitative evidence synthesis, qualitative systematic review

## Abstract

**Objective:**

The objective of this methodological scoping review was to investigate ways in which qualitative review teams are addressing equity, diversity, and inclusion (EDI) in the process of conducting and reporting qualitative systematic reviews that use JBI guidelines.

**Introduction:**

To promote health equity, there is a need for evidence synthesis processes and practices to develop approaches that incorporate EDI. Some guidance is available to guide equity-focused review methods and reporting, but this is primarily oriented to quantitative systematic reviews. There is currently limited knowledge about how review teams are addressing EDI within qualitative evidence syntheses.

**Inclusion criteria:**

This review included English-language qualitative systematic reviews, published in 2022, that used all the stjpg outlined in the JBI guidance for qualitative reviews.

**Methods:**

A 1-year sample of published reviews was identified from a search undertaken on March 17, 2023, of 2 health care databases: MEDLINE (Ovid) and CINAHL (EBSCOhost). Data extraction followed a framework approach, using an adapted pre-existing equity template. This included attention to i) the reporting of a range of characteristics associated with EDI, ii) search approaches, and iii) analytical approaches (including reflexivity, intersectionality, and knowledge user engagement). Data were analyzed using descriptive statistics and narrative summary.

**Results:**

Forty-three reviews met the inclusion criteria. The majority of the reviews (n = 30) framed their questions and aims in a generic/universal (rather than EDI-focused) way. Six reviews justified their population focus in terms of an EDI-related issue. Only 1 review included a knowledge user. The sociodemographic and other key characteristics of the samples in underpinning studies were poorly reported, making it hard to discern EDI-related issues or to undertake EDI-related analyses. Thirteen of the reviews included non-English-language evidence sources, and 31 reviews included gray literature sources. Ten reviews demonstrated an element of intersectional or otherwise critical approach within their analyses of categories and synthesized findings (whereby issues of power and/or representation were explicitly considered). Only 8 reviews included discussions of review team composition and reflexivity within the review process.

**Conclusions:**

This EDI-focused methodological enquiry has highlighted some limitations within current qualitative evidence synthesis practice. Without closer attention to EDI, there is a danger that systematic reviews may simply serve to amplify, rather than illuminate, existing gaps, silences, and inequitable knowledge claims based on dominant representations. This review sets out a range of suggestions to help qualitative evidence synthesis teams to more systematically embed EDI within their methods and practices.

**Review registration:**

Open Science Framework https://osf.io/wy5kv/

## Introduction

In recent years, there has been an increased focus on equity, diversity, and inclusion (EDI) within health research.^1^,^2^ In relation to health, equity reflects a concern for social justice whereby everyone can attain their full potential for health and well-being.^2^–^4^ When differences in health are unnecessary or avoidable, and considered unfair or unjust, they are considered to be health inequities.^5^,^6^ Reducing health inequities is an essential public policy objective.^7^ Efforts to promote equity are underpinned by concepts of equality, diversity, and inclusion. Equality refers to the provision of equal access to opportunities and resources, making sure that everyone is treated fairly, whereas equity acknowledges that the circumstances of a person or a group may differ, and so opportunities may need to be allocated differently to ensure an equal outcome. Diversity means promoting an environment and culture that welcomes and values diverse backgrounds, thinking, skills, and experience. Inclusion refers to processes through which all are treated with dignity and respect, and feel valued and accepted.^2^,^8^

With respect to research, there is increasing recognition that systemic biases exist within the research ecosystem; for example, in terms of funding allocations, question prioritization, research team composition, or research recruitment practices.^9^ It has been argued that much health-related research has historically excluded key populations or perspectives, perpetuating dominant worldviews and/or upholding unequal power relations.^10^ Thus, there is increasing recognition that attention to equality, diversity, and inclusion within the research process is necessary, and that research practices and outcomes should be oriented to the promotion of equity for health.^11^

Within the field of evidence synthesis, policymakers report that the lack of equity considerations in systematic reviews limits their usefulness for decision-making, and an increasing number of methodological investigations are demonstrating that systematic reviews pay insufficient (if any) attention to equity.^12^–^16^ The COVID-19 pandemic has further highlighted the urgent need for evidence syntheses to address equity.^17^

The Cochrane Equity Methods Group and the Cochrane Public Health Review Group have developed a framework titled PROGRESS-Plus,^16^ which sets out a range of intersecting characteristics that can influence health equity (see Table 1). These characteristics can be utilized within systematic review analyses to consider equity outcomes and processes more explicitly.^18^–^20^ The degree to which these factors are associated with disadvantage depends on time, place, and interaction between the factors.^21^

**Table 1:** PROGRESS-Plus characteristics

**PROGRESS** Place of residence Race/ethnicity/culture/language Occupation Gender/sex Religion Education Socioeconomic status Social capital
**PLUS (examples of additional characteristics), eg**, Personal characteristics associated with discrimination and/or exclusion (eg, age, sexual orientation, disability) Features of relationships (eg, abuse) Time-dependent relationships (eg, migration, just leaving hospital—where the person may be temporarily at a disadvantage)

Since 2012, a specific equity-focused systematic review reporting guideline (PRISMA-Equity Extension) has been developed to encourage reviewers to consider equity issues.^22^ PRISMA-Equity is designed to prompt reviewers to identify, extract, and synthesize evidence on equity in systematic reviews to improve the reporting of the effects of both inequities in health outcomes and health care use across the PROGRESS-Plus characteristics, thus contributing to the global agenda to improve health equity. More recently, Dewidar *et al.* 17 proposed a comprehensive framework for considering equity in relation to the key stjpg of a systematic review.

To date, efforts to apply an equity perspective in evidence synthesis have primarily focused on the methods and reporting processes within *quantitative* systematic reviews.^12^,^13^ Within these debates, some groups have suggested that greater use of *qualitative* systematic reviews (eg, as an adjunct to a quantitative review or as a mixed methods review) can also contribute to the equity agenda.^16^,^17^ For example, qualitative evidence synthesis (QES) can help to examine the contextual or EDI features that may influence intervention implementation, the differential experiences of those affected by interventions, and, potentially, the social and behavioral processes through which social power relations, exclusion, and inequalities manifest themselves in different ways to influence intervention outcomes. Until now, however, there has been little focus on how the methods and processes within QES *themselves* may need to take greater account of equity to maximize their potential contribution to the equity agenda. For example, to our knowledge, there are no QES reporting guidelines that explicitly incorporate equity considerations.^23^,^24^ In addition, there is a lack of knowledge of the extent to which qualitative review teams are currently taking EDI into account or of the different approaches they may be taking to address this issue.

This scoping review^25^–^27^ aimed to explore the ways in which QES teams are addressing EDI within their reviews. The current enquiry focuses on QES that have used methodological guidance from JBI. As one of the key global organizations in evidence-based health care, a core mission of JBI is to generate, synthesize, transfer, and implement evidence to promote global health, and to recognize and respect diversity as an integral part of that endeavor.^28^–^30^ JBI provides methodological guidance for systematic reviews, including for QES.^31^,^32^ The majority of authors on this paper are members of JBI Collaborating Centres and/or the JBI Qualitative Reviews Methodology Group. Hence, the intention was that this project would stimulate debate around EDI within the QES process.

As with quantitative reviews,^16^ the approach to equity within QES will depend on its aim and purpose. Some QES, for example, have a focus on an equity-related issue (either explicitly or implicitly), whereas others are focused on problems that are represented as relatively generic (see Table 2).

**Table 2:** Potential ways in which equity can be addressed within qualitative evidence synthesis

**Equity-related qualitative evidence synthesis (directly or indirectly)** Highlight an equity issue directly (eg, to highlight a diversity of experience according to different characteristics, to highlight the voices of an under-served or marginalized group or the experience of those in a disadvantaged setting) Help explain the equity-related mechanisms and outcomes of an intervention Help consider how an intervention could be appropriately transferred to different settings/populations groups, including low-income settings or vulnerable groups
**Not explicitly equity related** Address, explore, and illuminate a range of important health-related issues where equity or equity, diversity, and inclusion is not explicitly stated as a focus (but where these issues may nonetheless apply and are potentially being underexamined) or where data are not explicitly disaggregated

For QES that are equity-focused, it is important to note that, although the equity focus may be explicit, there may still be important differences in experiences or social processes related to PROGRESS-Plus characteristics, power, and resources *within* a group or setting. These differences may not always be obvious unless there is a sensitivity to them within the review process. For example, high-level data extraction about subpopulations, such as geographical location or ethnic background, may lack sufficient granularity to draw attention to the personal characteristics of research participants that may be associated with discrimination and/or exclusion.

One theoretical approach for enhancing such sensitivity to health equity is the concept of intersectionality.^2^ This has been defined by Crenshaw^33^ as “a metaphor for understanding the ways that multiple forms of inequality or disadvantage sometimes compound themselves and create obstacles that often are not understood among conventional ways of thinking.”^(p.149)^ Intersectionality can serve as a tool for understanding invisible power relations and how they shape inequality. By examining how interlocking systems of oppression play out in individuals’ lives, intersectional approaches to qualitative data analysis seek to uncover and theorize inequality within and between groups of people based on the ways in which multiple facets of an individual’s (or group’s) identity and disadvantage interact.^34^ Key attention is paid to observing cross- or intergroup patterns or variations in the data, paying particular attention to outliers (where the data does not seem to fit the theory or thematic patterns). To date, there is limited understanding of how intersectional analyses might best be approached within different types of QES.

Another approach to incorporating EDI within the QES process is to involve and engage knowledge users (patients and the public). This is increasingly recognized as a key feature of good research practice by ensuring that diverse voices and experiences are included, and that research questions and analyses include a sensitivity to the priorities, context, standpoint, and lived realities of all those affected by the research topic. The ways in which knowledge users are involved in QES can vary, however, ranging from a relatively tokenistic instrumental engagement to a community-led, co-produced, and participatory process in which there is an explicit focus on uncovering and challenging inequitable social power structures.^35^,^36^ Currently, there is little understanding about the ways in which knowledge user involvement is being used to enhance EDI specifically within QES.^37^

Reflexivity is another mechanism that can be employed to enhance sensitivity to EDI within qualitative research.^38^ Here, the research team engages in a variety of self-reflective practices to consider their own social identities, positions, values, assumptions, interests, and experiences in order to critically reflect on how these are shaping the research process and subsequent knowledge claims (including acknowledgment of prejudices, blind spots, and unnoticed framings).^39^ This process includes a consideration of power relations within the team itself, helping to uncover ways in which certain knowledge claims or positions may become privileged over others. Engaging in an analytical reflexive process and accounting for this in the research report is considered a hallmark of high-quality qualitative research.^39^,^40^ Questions related to reflexivity are found in all the major tools used to assess methodological quality of qualitative studies.^41^ For example, the JBI checklist for qualitative research^31^ has 2 questions related to reflexivity and the Critical Appraisal Skills Programme (CASP) tool^42^ has 1 question. The critical appraisal process within a QES is undertaken to enhance the review team’s sensitivity to the methodological strengths and weaknesses within the evidence base.

The JBI QES review process is underpinned by a pragmatic descriptive phenomenological approach in which meta-aggregation is the analytical strategy.^31^,^32^,^43^ It is well recognized that a danger with evidence synthesis, especially the more aggregative forms of QES, is that it can tend towards illuminating the general or average experience and, hence, nuance can be lost, making it challenging to retain an in-depth focus on context.^44^ Therefore, attention to equity may require further elaboration.

A preliminary search of PROSPERO, MEDLINE, the Cochrane Database of Systematic Reviews, *JBI Evidence Synthesis*, and Open Science Framework was conducted in March 2023. No current or in-progress systematic or scoping reviews on EDI processes within QES were identified.

The specific objectives of the project were to:

- explore and describe the ways in which EDI may have been addressed, thus providing a picture of the extent to which EDI is (or is not) being considered within qualitative reviews that have used JBI guidelines
- stimulate debate regarding ways in which equity can be addressed for different stages of the JBI qualitative review process.

## Review question

What are the ways in which qualitative systematic review teams are addressing health EDI in the process of conducting and reporting findings of qualitative systematic reviews that use JBI guidelines?

## Inclusion criteria

### Participants

This review included qualitative systematic reviews that explicitly stated that authors followed the full JBI qualitative guidelines and meta-aggregative approach, including the use of the ConQual approach to assess confidence in review findings.

### Concept

The concept in this review referred to the ways in which health equity was (or was not) addressed in the philosophy, process, methods, and findings of the included reviews.

### Context

This review included reviews related to any context or population (including qualitative reviews that have a specific equity focus and those that do not).

### Types of sources

Published qualitative systematic reviews conducted using full JBI guidelines were eligible for inclusion.

## Methods

This methodological review was conducted in accordance with the JBI methodology for scoping reviews, as it sought to map and summarize key features of a sample of evidence.^45^–^47^ The review is reported in line with the Preferred Reporting Items for Systematic Reviews and Meta-Analyses extension for Scoping Reviews (PRISMA-ScR).^48^ The review followed an a priori protocol, which is registered and publicly available at Open Science Framework.^49^

### Search strategy

The review adopted a convenience sampling approach, searching MEDLINE (Ovid) and CINAHL (EBSCOhost) for a 1-year period (2022). This date range provided a contemporary picture of review practice (given that the review was undertaken in 2023), while allowing the review to be completed within the time and resources available. The two databases were selected for pragmatic reasons in order to identify reviews that have been published in the JBI journal *JBI Evidence Synthesis* as well as reviews that have used the JBI approach but have been published in other sources.

The following keywords/MeSH were used in the search strategy: Review, Systematic review, Meta-synthesis, Metasynthesis, Evidence synthesis, Qualitative, JBI, Joanna Briggs Institute, Meta-aggregat*. The full search strategies for MEDLINE and CINAHL are presented in Appendix I. Searches were undertaken on March 17, 2023.

The inclusion and exclusion criteria were as follows: i) published in 2022, ii) must be published and peer reviewed (no gray literature), iii) full reviews (not protocols), iv) English language only, and v) must have followed the full JBI methodology (reviews that adapted or missed any part of the JBI methods guidance were excluded).

### Review selection

All identified records were collated and uploaded into EndNote vX9.3 (Clarivate Analytics, PA, USA) and duplicates removed. Potentially relevant studies were retrieved in full and their citation details were imported into Rayyan software (Qatar Computing Research Institute, Doha, Qatar).^50^ The full texts of potentially relevant records were assessed in detail against the inclusion criteria, with reviews excluded at this stage listed in Appendix II. All screening and selection processes were undertaken by 2 reviewers (ZH and CE), with recourse to the wider team in case of any disagreements.

### Data extraction

Data extraction was guided by the framework by Dewidar *et al.*^17^ for considering equity in relation to key systematic review stjpg. This framework formed the basis for the data extraction template (see Table 3 for a summarized version, with a full version in Appendix III). This framework was selected, as it builds in the principles of using PROGRESS-Plus^22^ characteristics to support intersectional/equity-focused analyses, as well as knowledge user involvement and team values/composition (reflexivity). It also highlights other potential equity-related issues, particularly in relation to the consequences and impacts of different choices related to key evidence sources (eg, limiting databases to English only or choices around the use of gray literature).^51^–^54^ The data extraction template included domains from the pre-existing framework^17^ (shaded in gray in Table 3), with 3 additional domains added (shaded blue in Table 3). Items extracted within each domain reflected the review team’s interpretation of i) what kind of data were important for the purpose of the study and ii) how the domain concept could specifically be applied to qualitative reviews. The full data extraction template in Appendix III includes more detail for each domain, as well as questions and prompts that were used to aid data extraction and subsequent analysis. The template was piloted with all team members, following which, data extraction and analysis were undertaken in Microsoft Excel (Redmond, Washington, USA) by ZH and CE. Areas of ambiguity that arose in data extraction were resolved through ongoing discussions with the review team.

**Table 3:** Summarized data extraction template

Data extraction domains	Data extraction items
Characteristics of the review	Citation details Review aim and objectives Geographical focus Population focus Number of included studies in the review
Engaging relevant knowledge users in conducting, designing, and interpreting the review	Knowledge user involvement (present or not; nature of contribution; stages of involvement)
Reflecting on equity in team values and composition	Identity/characteristics/composition of review team Reflexivity (descriptive and analytical)
Developing research questions to assess health inequities	Equity considerations within stated review aim and objectives
Identifying population(s) experiencing inequities	Equity considerations within stated geographic focus and population focus
Conducting searches in relevant disciplinary databases	Type and justification of databases Type and justification of gray literature sources Inclusion/exclusion of languages other than English
Collecting data for equity	PROGRESS-PLUS dimensions related to samples in included papers
Analyzing evidence on equity	Critical appraisal (reporting and reflection on reflexivity and reporting and reflection on EDI within included studies) Attention to EDI within approaches to synthesis (eg, subgroup or sensitivity analyses, analyses of intersectionality, analyses attending to issues of power and representation)
Evaluating the applicability of the findings to populations experiencing inequities or other settings	Confidence in the review findings (ways in which EDI considerations may influence ConQual assessments) Discussion (eg, are EDI considerations addressed?) EDI considerations influencing transferability to the context of the review question Considerations of transferability to populations or contexts of disadvantage or under-representation
Adhering to reporting guidelines for communicating review findings	Not extracted/analyzed further, as all included reviews adhered to full JBI reporting guidelines
Reflections on review strengths and limitations	Identification of EDI issues influencing the review process and conduct (links to reflexivity)
Reflections on EDI within review recommendations	Inclusion of recommendations related to EDI

Gray shading: domains used from framework proposed by Dewidar, *et al.* 2022.^17^

Blue shading: domains added by the review team.

EDI, equity, diversity, and inclusion.

### Data analysis and presentation

The data were summarized using descriptive statistics and narrative summary.

### Review team and reflexivity

The review team comprised a multiprofessional (nursing, medicine, public health) and multidisciplinary (health science, social science) group of researchers at different career stages and of different cultural and ethnic backgrounds. As a team, our familiarity with EDI concepts and discourse varied considerably; we engaged in regular reflexive discussions and felt that we were on a collective learning journey. Through this reflexive process, there was recognition that, although we prioritize EDI in our primary research practice, we have not always translated this to QES. Our stance throughout this enquiry was, therefore, not to critique individual authors but to illuminate current QES practice through an EDI lens. The results of our study are thus presented descriptively and without judgment. Likewise, our subsequent discussion and recommendations are not focused on what was reported or omitted in individual reviews, but rather on how QES guidance can encourage review teams to make equity-related considerations more explicit in future.

## Results

### Review inclusion

**Figure 1:**
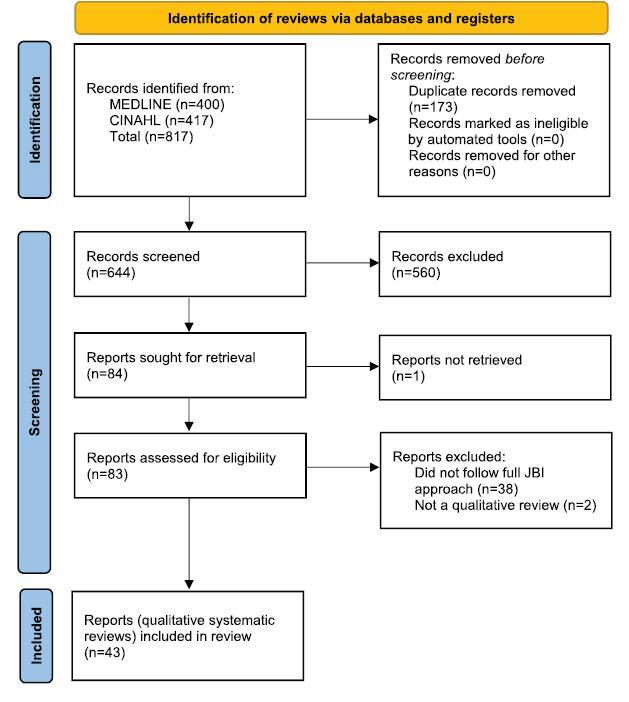
Search results and review selection and inclusion process^98^

Following de-duplication, the searches identified 644 records, of which 84 were identified as potentially eligible for inclusion based on their title and abstract. Of these, 1 paper could not be retrieved. Of the remaining 83 records, 40 were excluded (see Appendix II): 2 were not qualitative reviews and the others did not apply the full JBI approach^32^ (primarily not applying the ConQual assessment of confidence in the review findings). Forty-three reviews met the inclusion criteria and were included in the review.^55^–^97^ The search process and results are documented in a PRISMA flow diagram (Figure 1).^98^

### Characteristics of included reviews

Full details of the included reviews, including their aims and objectives, are outlined in Appendix IV. The number of included studies within the reviews ranged from 3 to 57.

### Review findings

The findings are reported according to the domains of the data extraction template (as per Table 3). The analysis focused solely on what was reported in the reviews. However, we are aware that absence of *reporting* does not necessarily relate to absence of *doing*, and that depth of reporting can also be constrained by journal word count restrictions.

#### Engaging knowledge users

Only 1 of the included reviews reported any kind of knowledge user involvement in the process of the systematic review.^88^ In this example, the knowledge user was a health professional experienced in the field of tobacco control and smoking cessation counseling, and was involved in a validation exercise undertaken at the end of the review process.^88^

#### Reflecting on equity in team values and composition

Eight of the included reviews provided some commentary of team composition and reviewer identity, but these were generally brief descriptive statements.^60^,^63^,^67^,^71^,^74^,^77^,^87^,^88^ Four of these accounts included descriptions related to positionality.^63^,^71^,^74^,^77^ For example, May *et al.* 77 reported that the review team had credentials and expertise in research and in the subject area. The authors stated that, in keeping with quality standards for rigor in qualitative research, they had considered their theoretical positions, views, and opinions on the topic and any possible influence this would have on the review.^77^ In another example, Lim *et al.* 74 reported that the primary reviewer practiced reflexivity by keeping a journal detailing their reflections during immersion in data analysis and that 2 of the authors engaged in reflexive discussions. Only 3 reviews described a more in-depth analytic consideration of reflexivity.^67^,^71^,^74^ These included, for example, how the authors had engaged in robust discussion throughout the review process in relation to their own standpoints, experiences, and perspectives, and how these may have related to the analytical process or the stjpg that were taken to minimize the authors’ preconceptions influencing the research process.

#### Developing research questions to assess health inequities

The majority of the included reviews (30/43; 69.8%) framed their aim as a relatively generic/universal issue (eg, experiences of self-management in breast cancer survivors with lymphedema,^66^ or barriers and enablers to physical activity participation in people with venous leg ulcers^86^). Nine of the included reviews had an aim that was more explicitly focused on an EDI-related issue,^60^,^62^,^64^,^68^,^71^,^79^,^85^,^87^,^90^ for example, by focusing on country income level (low- and middle-income countries [LMICs]),^64^ gender disparities, or the impact of low socioeconomic status.^79^

#### Identifying populations experiencing inequities

The majority of the included reviews (36/43; 83.7%) had a global geographical focus (ie, including studies from any country context). Seven (16.3%) of the included reviews focused on more specific geographical locations such as sub-Saharan Africa,^56^,^90^ other LMICs,^64^ high-income countries,^61^–^63^ or Middle Eastern countries.^68^ Of these, 5 reviews linked the rationale for the more specific geographical context to equity.^56^,^63^,^64^,^68^,^90^ For example, Egan *et al.* 64 sought to highlight barriers and facilitators to educational access and excellence for students with disabilities, focusing specifically on this phenomenon in resource-poor settings (ie, in African countries).

The included reviews had a wide variation of population focus, depending on their different aims. Six reviews justified their choice of population specifically in relation to EDI concerns, such as investigating the experiences of a specific gender with a disease, highlighting exclusion experiences associated with disability, or analyzing experiences in relation to socioeconomic status.^60^–^62^,^64^,^79^,^87^

#### Conducting searches in relevant disciplinary databases

Regarding the search strategies carried out in the included reviews, nontraditional database sources were considered in 30.2% (13/43). Examples included African Index Medicus, ATLA Religion Database, Japan Medical Abstracts Society, CNKI (Chinese), and Latin American and Caribbean Centre on Health Sciences Information.^56^,^57^,^68^,^72^,^73^,^75^,^79^–^81^,^87^,^89^,^94^,^97^ Furthermore, more than one-third (15/43; 34.9%) of the included reviews considered non-English sources such as French, Spanish, or Chinese.^56^,^57^,^68^,^72^,^73^,^75^,^76^,^79^,^81^,^87^–^89^,^91^,^92^,^97^ Gray literature was not searched in 27.9% of the included reviews (12/43).^55^,^58^,^59^,^66^,^67^,^70^,^73^,^79^,^81^,^83^,^89^,^94^

#### Collecting data for equity

The reviews were examined to consider the ways in which the sample characteristics of their included studies were reported in relation to the PROGRESS-Plus framework. All the included reviews reported the countries where their included studies were undertaken. Seven reviews reported the gender of the participants in their studies,^56^,^57^,^60^,^62^,^71^,^72^,^87^ and 16 reviews reported the age of the participants in their studies.^59^,^61^,^64^,^72^–^75^,^80^,^86^–^90^,^92^,^94^,^96^ Seven reviews reported on the specific geographical or cultural context, such as sub-Saharan Africa, Middle Eastern countries, LMICs or high-income countries.^56^,^61^–^64^,^68^,^90^

A limited number of included reviews reported broader details about sociodemographic characteristics of participants in their studies, such as occupation (n = 9; 20.9%),^61^,^63^,^76^–^78^,^87^,^88^,^91^,^93^ living in rural or remote areas (n = 3; 7%),^63^,^65^,^93^ having a disability (n = 3; 7%),^59^,^64^,^87^ ethnicity (n = 3; 7%),^71^,^72^,^87^ culture (n = 1; 2.3%),^87^ socioeconomic status (n = 1; 2.3%),^87^ language (n = 2; 4.7%),^87^,^88^ migration status (n = 1; 2.3%),^71^ or relationship status (n = 1; 2.3%).^88^

Data related to religion and sexual orientation were not reported in any of the included reviews.

#### Analyzing evidence on equity

With respect to the critical appraisal process undertaken by review teams, the narrative around critical appraisal in almost all included reviews drew attention to the phenomenon of reflexivity within the included papers as an element of their assessment of methodological quality. However, they primarily did this by reporting factually on their evaluation of questions 6 and 7 on the JBI Checklist for Qualitative Research; for example, by stating the number of studies that were deemed to have “adequately located the researcher either culturally or theoretically” (Q6) or the number of studies that had “stated the influence of the researcher on the research” (Q7). Twelve (27.9%) of the included reviews provided more in-depth critical commentary related to their stance on the relative importance of different quality criteria^56^,^62^,^71^,^76^–^78^,^80^,^87^,^89^,^90^,^93^,^97^ (eg, how this influenced their views on the overall quality of the body of evidence^90^ or whether to include or exclude studies that scored poorly on certain criteria^71^).

Two of the included reviews reflected critically on the limitations of their included studies in terms of equity or PROGRESS-Plus characteristics.^59^,^71^ For example, Kassam *et al.* 71 commented on the gender, education, and social status of the participants and the extent to which the included papers had (or had not) considered intersectionality. Conti *et al.* 59 mentioned that it was not possible to consider the gender, education, or social status of participants, as they were not stratified in the primary studies.

In terms of data synthesis, none of the included reviews undertook a subgroup or sensitivity analysis (although this is to be expected, as it is not part of current JBI guidance). Ten (23.3%) of the included reviews demonstrated an element of intersectional or otherwise critical approaches within their analyses of categories and synthesized findings (whereby issues of power and/or representation were explicitly considered).^60^,^62^,^64^,^65^,^68^,^71^,^79^,^85^,^87^,^90^ For example, Egan *et al.*,^64^ Emmett *et al.*,^65^ Hassanein *et al.*,^68^ and Tanywe *et al.* 90 explored the potential intersections of the geographical location and/or cultural beliefs on the experience of health conditions, perceptions of risk, access to health facilities, or other medical services (eg, access of disabled students to medical services in Africa, the impact of sociocultural beliefs on the management and perception of risk of trachoma in resource-poor African settings). Meng *et al.* 79 reported that patients with advanced cancer who were of low socioeconomic status experienced unaffordable medical care resulting in delays in care-seeking, noting that patients with different cultural backgrounds were more severely impacted due to being less able to have adequate conversations with health care providers. In another example, Kassam *et al.* 71 reported that migrant pregnant women experienced racism, discrimination, isolation, fear, and uncertainty related to the future.

#### Evaluating the applicability of the findings to populations experiencing inequities or other settings

One element of this domain relates to ways in which EDI considerations may influence the assessment of confidence in the review findings. To consider this, the project team examined ways in which reflexivity (as assessed within the underpinning included studies) may have influenced ConQual assessments within the included reviews. This is because questions 6 and 7 of the JBI Checklist for Qualitative Research form part of ConQual’s assessment of dependability in relation to synthesized findings. Thirty-one (72.1%) of the 43 included reviews explicitly mentioned that their ConQual assessment process was influenced by their assessment of methodological limitations related to reflexivity.^55^,^57^–^59^,^63^–^67^,^71^–^78^,^82^,^83^,^85^–^88^,^90^–^97^ Specifically, the ConQual dependability assessment for certain synthesized findings in these reviews was downgraded due to poor reporting of questions 6 and 7 on the JBI Checklist for Qualitative Research in their underpinning studies.

EDI considerations featured in various ways in the discussion sections of the included reviews depending on the nature of the review question and objectives. The majority of the included reviews included a reflection on the potential transferability of their findings to other populations or contexts.^57^–^59^,^62^–^65^,^67^,^71^–^76^,^79^–^81^,^83^,^86^–^88^,^90^–^93^,^95^–^97^ Reflections on transferability were often related to similarities or differences of the contexts of the underpinning studies. For example, Chang *et al.*^58^ reported that the majority of the studies included in their review were from the UK, meaning that findings might not be applicable to countries with different health systems, cultures, or environments. Conversely, Casaleiro *et al.*^57^ reported that their systematic review drew on data from different geographical areas, cultures, and religious backgrounds, thus, it was possible to identify similarities in spite of the diverse contexts, and the findings could be transferable across different contexts.

To a lesser degree, some discussion/conclusion sections of the reviews suggested insights on overarching structural issues that may influence transferability of the review findings. For example, some reviews highlighted structural barriers to access to resources (eg, noting how geographical location could affect access to health care^55^ or access to education^64^). Others reflected on the impact of socioeconomic disparities on access to health services.^70^,^79^,^82^ Some reviews reflected on ways in which social and gender norms could affect ability to adopt healthy lifestyles^56^ or access support.^62^ Other reviews discussed issues related to social exclusion (eg, associated with disability^72^,^73^,^87^,^89^) and discrimination (eg, ageism^74^).

#### Reflections on review limitations and strengths

Within the limitations sections of the included reviews, several reflected on EDI-related issues as potential limitations. For example, 15 reviews recognized that there may have been some selection bias within the body of evidence, as only English-language papers had been included.^58^,^60^,^61^,^63^–^66^,^69^,^71^,^78^,^80^,^82^,^83^,^86^,^90^ Five reviews also recognized this issue in relation to not having included gray literature in their search strategy.^66^,^67^,^73^,^81^ Four of the included reviews noted potential issues relating to the paucity of nuance or specificity in the underpinning evidence related to their topic.^64^,^68^,^71^,^83^ For example, Abdul Rahman *et al.* 55 reported that all of the studies in their review (which aimed to explore the experiences of patients with leprosy), had explored the impact of leprosy on women, with no studies having included men. Thus, the authors recommended that this bias could potentially be investigated in future research.^55^ Kassam *et al.* 71 mentioned that within the studies identified in their review (on the experiences of nurses caring for involuntary migrant maternal women), there was minimal mention of ethnicity and gender as health determinants. The authors of that review noted that this limitation surfaced through the application of the team’s theoretical standpoint on intersectionality (in which they saw social variables including gender, ethnicity, and migrant status interacting in complex ways to generate inequitable impacts).^71^

#### Reflections on equity, diversity, and inclusion within review recommendations

Many of the included reviews drew attention to issues related to EDI within the recommendations section of their reports. Reflections by review authors related to recommendations for action or improved sensitivity to EDI in the context of i) review/research methods, ii) recommendations for future research needs, and iii) recommendations for policy and practice. Some examples of these are provided in Table 4.

**Table 4:** Examples of review recommendations related to equity, diversity, and inclusion

**Examples of EDI-related recommendations for review/research methods**
A need to address selection bias in the review process (eg, inclusion of non-English-language articles in reviews^55^,^97^) A need for better reporting of qualitative studies (eg, Zhu *et al.* 96 recommended that the original qualitative studies should report their context, methodology, philosophical foundation, and researcher reflexivity more clearly)
**Examples of EDI-related recommendations for future research studies**
A need for inclusion of under-represented groups, countries, or settings in future research and recommendations related to the need for intersectional analyses within future research (eg, Davenport *et al.*,^62^ who explored fathers’ experiences of depression in the perinatal period, recommended that research is needed to better represent the experiences of fathers from more diverse sociodemographic backgrounds, particularly those backgrounds that have been historically marginalized and/or rendered invisible in the academic literature; racial minority fathers; and possibly different experiences between first-time and subsequent fathers)
**Examples of EDI-related recommendations for policy/practice**
A need for recommendations on how to address causes of inequality, disadvantage, stigma, or exclusion highlighted within the review findings (eg, Cooper-Stanton *et al.* 60 recommended the involvement of men within the design and commissioning of lymphoedema services to ensure that their needs are recognized and addressed; Tanywe *et al.* 90 recommended that decision-makers need to consider the sociocultural and economic barriers to the effective adoption of preventive behaviors when designing interventions to eliminate blinding trachoma; Meng *et al.* 79 recommended that health care professionals should ensure they attend to the specific needs of low-socioeconomic-status individuals with cancer, develop effective communication with them, and help formulate effective strategies to provide support; Meng *et al.* 79 also recommended that governments should develop appropriate policies to ensure daily necessities and care of this vulnerable group)

EDI, equity, diversity, and inclusion.

## Discussion

A recent JBI position paper states that “working with clinical and academic experts in universities and health facilities from all health professions across the world ensures that the research evidence we seek to synthesize, transfer and implement is culturally inclusive and relevant across the diversity of healthcare internationally.”^29^^(p.192)^ This methodological review aimed to characterize the ways in which qualitative review teams are currently addressing EDI within their reviews and associated methodological processes. The enquiry is particularly salient, as an analysis undertaken in 2022 estimated that QES currently comprise 22.5% of all reviews published in *JBI Evidence Synthesis* (compared, for example, with 0.19% of reviews published in the Cochrane Database of Systematic Reviews, n = 17).^99^

Overall, this methodological review found that EDI is currently not being addressed in an explicit or systematic way in the context of QES. In order to stimulate further debate in this area, we suggest that 2 overlapping issues may benefit from further methodological investigation: i) incorporating an EDI focus within review methods, and ii) adopting EDI-related research practices within a review.

### Incorporating an EDI focus within review methods

As described in the previous sections, sensitivity to EDI can be built into the standard stjpg of a qualitative systematic review process. Arguably, the most important of these is the review question. The findings of this review show that the majority of QES that used the JBI approach did not explicitly mention EDI-related issues as part of their rationale for framing the review question or for the subsequent inclusion/exclusion criteria. Rather, in most cases, both population and geographical context tended to be framed in a universal way. The impact of framing a review as a multicontext (universal) vs single-context (more specific) enquiry (sometimes referred to as lumping or splitting) is an area that requires further research.^100^,^101^ Decisions about question-framing have implications in terms of the authority of the knowledge claims of a review (currently assessed by ConQual^102^), and also relate to the potential transferability of review findings. We note that ConQual does not currently incorporate a dimension related to questions of transferability.^102^ However, our review found that the majority of review authors were rightly cautious and nuanced when discussing transferability of their synthesized findings.

Another key review step relates to locating relevant sources of evidence. The majority of QES in the study sample searched for gray literature. This recognizes that important evidence may be found outside of traditional authoritative sources that can exclude certain communities, ways of knowing, or ways of sharing knowledge.^53^,^54^,^103^ However, only one-third of reviews included non-English-language databases (in spite of the majority of reviews having a global framing). There is an ongoing need to consider how best to incorporate languages other than English into a review.^104^ JBI is at the forefront of innovations to address this issue, drawing on the resources of its global collaboration.^30^,^105^ Going forward, it will be important to evaluate the impact that greater inclusion of non-English-language sources may (or may not) have on QES findings.

The review findings suggest that there is considerable variation in relation to how QES review teams currently extract and report features of their underpinning evidence in relation to the PROGRESS-Plus^16^ characteristics (see Table 1). Lack of detail regarding these characteristics means that it is not always clear whose voices or which contexts are being represented (or not) within a review. Potential implications of this are that the experiences of a dominant group may come to be seen as representative of a phenomenon. Additionally, the synthesis product may fail to make clear where there are gaps in knowledge of how others may be experiencing a phenomenon, or how contextual factors may be influencing the phenomenon.^106^,^107^ This has potentially significant implications when using QES as part of clinical guideline development.^108^ Lack of detailed reporting on the PROGRESS-Plus^16^ characteristics is undoubtedly linked to a concomitant lack of detail within the underpinning studies of a review. However, by not explicitly and systematically considering EDI within the underpinning study samples, it remains unclear whether more detail was available but was not reported by the review team.

A relatively small proportion of reviews explicitly uncovered issues related to power or representation within their analyses. This may be linked to the framing of the review question (as multi- or single-context) or to a lack of information on PROGRESS-Plus^16^ characteristics in the underpinning evidence. It may also be linked to the descriptive phenomenological meta-aggregative approach of JBI reviews.^31^,^32^ This approach tends to seek commonalities of meaning and is limited in the extent to which context or patterns in the data can be explored in-depth.^44^ Given this methodological stance, we suggest that reviews seeking to be sensitive to EDI are better aligned to research questions that are focused on highly specific contexts or populations, rather than adopting a generic or universal approach.^44^ More research is needed on how an intersectional perspective might be included in a methodologically coherent way within a meta-aggregative synthesis.^44^ Abrams *et al.* 39 offer some useful pointers, suggesting that researchers can begin by asking themselves critical questions to enhance sensitivity to EDI while searching for common meanings, eg, “What commonalities exist across the multiple identities of participants?”^(p.4)^ Likewise, they note that:

After identifying participants of interests and their intersecting identities researchers should then critically examine the role of marginalization and the social forces that drive inequities as it relates to the phenomena under study … identifying how commonalities differ among certain intersectional identities that share a common axis (e.g., gender…) can aid researchers in exploring how barriers or facilitators are differentially efficacious among…sub-groups. 39^ (p.9)
^

When considering confidence in the synthesized findings of a review, we note that the JBI ConQual approach places a strong emphasis on assessment of methodological quality of underpinning studies.^102^ In relation to EDI, reflexivity gives an insight into the researchers’ identity, positionality, standpoint, and influence on the research process, which, in turn, enables reviewers to form more comprehensive assessments of study findings. The current review has found that the majority of review teams reported in a factual and descriptive way on this important issue (ie, factually describing the results of questions 6 and 7 on the JBI checklist for qualitative research). However, the majority of review teams did not provide any further critical commentary on the potential implications (for EDI or otherwise) of poor reflexivity in their underpinning body of evidence. The JBI ConQual approach means that synthesized findings based on studies that score poorly on reflexivity questions (among others) may be downgraded. Nonetheless, the specific EDI-related implications of this downgrading were rarely discussed.

#### Implications for review methods

Overall, our findings indicate 6 areas that may benefit from further methodological work to support the incorporation of EDI within review methods: i) framing the question (eg, exploring when and how to frame the population or context in a universal or highly specific manner); ii) considering approaches for inclusion of gray literature sources and studies in languages other than English; iii) considering optimal ways to describe the population, geographical, and other PROGRESS-Plus-related characteristics of the underpinning studies and considering how to highlight potential gaps in representation; iv) considering whether and how to address EDI and intersectionality within analyses; v) considering approaches to reflect on the nature of reflexivity in the underpinning evidence (and its potential impact on an understanding of EDI in relation to the phenomenon of interest); and vi) considering ways to reflect on the potential transferability of findings in relation to groups or contexts that were not represented in the underpinning evidence for the review.

### Adopting EDI-related research practice

The previous discussion relates to incorporation of EDI within the different procedural stjpg of a review. In this section, we consider the review team itself and its values and research practices in relation to EDI. We do this by considering 2 processes (reflexivity and knowledge user involvement) that aim to make research more transparent, more democratic, and, thus, more accessible to, and relevant for, policy and practice.^109^

In terms of reflexivity, the review found that only 8 reviews provided information on the review team’s identity or positionality, and only 3 of the 8 review teams offered a more detailed consideration of their standpoint or ways in which reflexivity influenced the review process. This is perhaps not unexpected, as JBI does not currently provide explicit guidance on incorporating reflexivity into the review process.^32^ In addition, we recognize that journal word counts may also limit review authors’ capacity for including reflexive statements in their manuscripts.^110^

JBI QES are not alone in poor reporting of reflexivity, however, as several authors have noted the same phenomenon in QES more generally, regardless of approach.^44^,^111^ Currently, the most commonly used reporting guideline for QES, ENTREQ, does not include reflexivity as a specific reporting criterion^24^; however, more recent reporting guidance is beginning to include this criterion (eg, the eMERGe Meta-Ethnography reporting guidance^23^ and guidance on undertaking a qualitative review produced by the former Cochrane EPOC group^112^). In addition, the most recent (2024) edition of the Cochrane/Campbell Qualitative Methods Handbook has, for the first time, included a section on reflexivity (and EDI) in each chapter.^113^

In relation to knowledge user involvement, only 1 review included knowledge users in their processes. We recognize that time or resource constraints can hinder meaningful involvement of knowledge users, especially for student projects. Nonetheless, given the importance and potential contribution in relation to EDI that knowledge users bring to evidence synthesis at all stages,^17^,^53^,^54^,^114^ we suggest that there is room for improvement in this area.

#### Implications for review practices

Going forward, we suggest that further consideration is given to the ways in which QES practices can incorporate a reflexive approach and include a critical reflexive statement. In relation to EDI, further work could consider how best to articulate theoretical standpoint, identities of team members, and potential power relations within the team.^38^,^115^ In line with JBI’s descriptive phenomenological methodological approach, the reflexive process may also involve a discussion of bracketing (ie, how the research team attempted to make explicit, yet avoid undue influence of, their own standpoints on the analytical process).^116^ Further guidance on reflexivity reporting may be helpful.

Likewise, we suggest that additional guidance may be helpful in relation to knowledge user involvement in QES. This could include support for review teams to consider their stance to knowledge user involvement in terms of equality (opening up spaces for all voices to be heard) and equity (attending to research and team practices relating to power and decision-making within the review process, and, potentially, the wider research ecosystem).

### Strengths and limitations

This innovative methodological scoping review sought to explore the ways in which EDI may be incorporated within QES. As a relatively novel area of methodological enquiry, we recognize a range of potential limitations of our approach. First, we investigated a relatively small sample of reviews (over a 1-year time period only). This approach means we are able to infer insights based only on this snapshot, but we have no reason to believe that other sampling approaches would have significantly altered the overall conclusions. Second, this review focused only on QES that used the JBI approach. Within the time and resource constraints of the current project, this was all that was possible. In the future, to develop guidance, it will be important to investigate how EDI is approached within a wider range of QES approaches. Third, we recognize an irony that, in spite of our critiques, our own project did not involve knowledge users or papers in languages other than English. This was due to time and resource constraints. Future methodological initiatives should endeavor to do so. Nevertheless, we hope that this review offers an initial starting point for further conversations and developments around EDI in QES.

## Conclusion

Our EDI-focused methodological enquiry has highlighted some limitations within current QES practice. Without closer attention to EDI, there is a danger that systematic reviews may simply serve to amplify, rather than to illuminate, existing gaps, silences, and inequitable knowledge claims based on dominant representations. Our study offers some initial suggestions that may help QES teams to more systematically embed EDI within their methods and practices. In doing so, we hope that review outputs will be better able to address questions of health equity. The JBI Qualitative Reviews Methodology Group is developing further guidance related to QES and EDI. Going forward, we suggest that updates of other reporting guidelines, such as PRISMA-Equity,^22^ consider QES in more detail.

## Acknowledgments

Our colleagues from the JBI Qualitative Reviews Methodology Group for supporting this project.

## Funding

The School of Health Sciences, University of Nottingham, provided a small grant to help to undertake this work.

## Author contributions

Conceptualization: CE, CB, DE, AE, M Bains, ZMH. Data curation: CE, ZH. Formal analysis: CE, CB, DE, AE, M Bains, ZMH, KP, M Bjerrum, SS. Funding acquisition: CE. Investigation: CE, ZMH. Methodology: CE, CB, DE, AE, M Bains, ZMH. Project administration: CE, ZH. Writing – original draft: CE, CB, DE, M Bains, AE, ZMH. Writing – editing and reviewing: CE, CB, DE, AE, ZMH, KP, M Bains, M Bjerrum, SS.

## Availability of data, code, and other materials

All relevant data are included in the report and its appendices. Any additional data can be obtained upon request from the corresponding author.

## Appendix I: Search strategy

### MEDLINE (Ovid)

**Table:** 1946–March 17, 2023

	Search term	Records retrieved
1.	systematic review.mp. or exp “Systematic Review”/ or Review Literature as Topic/ or (systematic adj2 review*).mp. or Review.mp. or Meta-synthesis.mp. or Metasynthesis.mp. or Evidence synthesis.mp.	3,948,219
2.	exp Qualitative Research/ or Qualitative.mp.	333,427
3.	JBI.mp. or Joanna Briggs Institute.mp. or JBI evidence synthesis.mp. or Meta-aggregation.mp. or meta aggregat*.mp.	5251
4.	1 AND 2 AND 3	1303

**Table:** With 2022 year limit

	Search term	Records retrieved
1.	systematic review.mp. or exp “Systematic Review”/ or Review Literature as Topic/ or (systematic adj2 review*).mp. or Review.mp. or Meta-synthesis.mp. or Metasynthesis.mp. or Evidence synthesis.mp.	3,948,219
2.	exp Qualitative Research/ or Qualitative.mp.	333,427
3.	JBI.mp. or Joanna Briggs Institute.mp. or JBI evidence synthesis.mp. or Meta-aggregation.mp. or meta aggregat*.mp.	5251
4.	1 AND 2 AND 3	1303
5.	limit 4 to yr = “2022”	400

### CINAHL (ESBCOhost)

**Table:** 1961– March 17, 2023

	Search term	Records retrieved
S4	S1 AND S2 AND S3	1950
S3	TX JBI or TX JBI evidence synthesis or TX Joanna Briggs Institute or TX meta-aggregation or (MH” meta-aggregation”)	8173
S2	(MH” qualitative studies”) or TX qualitative studies or TX qualitative	206,887
S1	TI ((Systematic N2 review*) or (MH” systematic review”) or TX systematic review or AB ((Systematic N2 review*) or “Meta-synthesis” or “Metasynthesis” or “evidence synthesis”	322,016

**Table:** With 2022 time limit

	Search term	Records retrieved
S4	S1 AND S2 AND S3	417
S3	TX JBI or TX JBI evidence synthesis or TX Joanna Briggs Institute or TX meta-aggregation or (MH” meta-aggregation”)	1296
S2	(MH” qualitative studies”) or TX qualitative studies or TX qualitative	23,560
S1	TI ((Systematic N2 review*) or (MH” systematic review”) or TX systematic review or AB ((Systematic N2 review*) or “Meta-synthesis” or “Metasynthesis” or “evidence synthesis”	31,958

## Appendix II: Reviews ineligible following full-text screening

**Table:**

Record	Reason for exclusion
1. Alnaeem MM, Bawadi HA. systematic review and meta-synthesis about patients with hematological malignancy and palliative care. Asian Pacific Journal of Cancer Prevention 2022; 23: 2881-90	Did not fully apply the JBI approach
2. Bulndi LB, Ireson D, Adama E, Bayes S. Sub-Saharan African women’s views and experiences of risk factors for obstetric fistula: a qualitative systematic review. BMC Pregnancy and Childbirth 2022; 22: 680	Did not fully apply the JBI approach
3. Chen W, O’Bryan CM, Gorham G, Howard K, Balasubramanya B, Coffey P, *et al.* Barriers and enablers to implementing and using clinical decision support systems for chronic diseases: a qualitative systematic review and meta-aggregation. Implementation Science Communications 2022; 3: 81	Did not fully apply the JBI approach
4. Collins J, Lizarondo L, Taylor S, Porritt K. Adult patient and carer experiences of planning for hospital discharge after a major trauma event: a qualitative systematic review. Disability and Rehabilitation 2022: 1-21	Did not fully apply the JBI approach
5. Diniz AMB, Manso PH, Santos MV, Rodrigues AJ, Sbragia L. A systematic review of benefits and risks of fetal surgery for congenital cardiac defects such as pulmonary valve stenosis and critical aortic stenosis. Brazilian Journal of Cardiovascular Surgery 2022	Was not a qualitative systematic review
6. Easton C, Oudshoorn A, Smith-Carrier T, Forchuk C, Marshall CA. The experience of food insecurity during and following homelessness in high-income countries: a systematic review and meta-aggregation. Health and Social Care in the Community 2022; 30: e3384-e3405	Did not fully apply the JBI approach
7. Fernandes ACNL, Palacios-Cena D, Pena CC, Duarte TB, de la Ossa AMP, Jorge CH. Conservative non-pharmacological interventions in women with pelvic floor dysfunction: a systematic review of qualitative studies. BMC Women’s Health 2022; 22: 515	Did not fully apply the JBI approach
8. Fielding C, Bramley L, Stalker C, Brand S, Toft S, Buchanan H. Patients’ experiences of cannulation of arteriovenous access for haemodialysis: a qualitative systematic review. The Journal of Vascular Access 2022: 11297298211067630	Did not fully apply the JBI approach
9. Firouzkouhi M, Abdollahimohammad A, Rezaie-Kheikhaie K, Mortazavi H, Farzi J, Masinaienezhad N, *et al.* Nurses’ caring experiences in COVID-19 pandemic: a systematic review of qualitative research. Health Sciences Review, 2022; 3: 100030	Did not fully apply the JBI approach
10. Goddard G, Oxlad M. Caring for individuals with type 1 diabetes mellitus who restrict and omit insulin for weight control: evidence-based guidance for healthcare professionals. Diabetes Research and Clinical Practice 2022; 185: 109783	Did not fully apply the JBI approach
11. Hamed MMM, Konstantinidis S. Barriers to incident reporting among nurses: a qualitative systematic review. Western Journal of Nursing Research 2022; 44: 506-23	Did not fully apply the JBI approach
12. Hyvamaki P, Kaariainen M, Tuomikoski A-M, Pikkarainen M, Jansson M. Registered nurses’ and medical doctors’ experiences of patient safety in health information exchange during interorganizational care transitions: a qualitative review. Journal of Patient Safety 2022; 18: 210-24	Did not fully apply the JBI approach
13. Ivziku D, Gualandi R, Pesce F, De Benedictis A, Tartaglini D. Adult oncology patients’ experiences of living with a central venous catheter: a systematic review and meta-synthesis. Supportive Care in Cancer 2022; 30: 3773-91	Did not fully apply the JBI approach
14. Jensen ED, Poirier BF, Oliver KJ, Roberts R, Anderson PJ, Jamieson LM. Childhood experiences and perspectives of individuals with orofacial clefts: a qualitative systematic review. The Cleft Palate-Craniofacial Journal 2022: 10556656221084542	Did not fully apply the JBI approach
15. Karacsony S, Merl H, O’Brien J, Maxwell H, Andrews S, Greenwood M, *et al.* What are the clinical and social outcomes of integrated care for older people? A qualitative systematic review. International Journal of Integrated Care 2022; 22: 14	Did not fully apply the JBI approach
16. Lao Y, Chen X, Zhang Y, Shen L, Wu F, Gong X. Critical care nurses’ experiences of physical restraint in intensive care units: a qualitative systematic review and meta-synthesis. Journal of Clinical Nursing 2023; 32: 2239-51	Did not fully apply the JBI approach
17. Leighton K, Kardong-Edgren S, McNelis A, Sullo E. Learning Outcomes Attributed to Prelicensure Clinical Education in Nursing: A Systematic Review of Qualitative Research. Nurse Educator 2022; 47: 26-30	Did not fully apply the JBI approach
18. Li C, Zhou Y, Zhou C, Lai J, Fu J, Wu Y. Perceptions of nurses and physicians on pay-for-performance in hospital: a systematic review of qualitative studies. Journal of Nursing Management 2022; 30: 521-34	Did not fully apply the JBI approach
19. Liu S, Duan X, Han P, Shao H, Jiang J, Zeng L. Occupational benefit perception of acute and critical care nurses: a qualitative meta-synthesis. Frontiers in Public Health 2022; 10: 976146	Did not fully apply the JBI approach
20. McKie AL, Turner M, Paterson C. What are the qualitative experiences of people affected by kidney failure receiving haemodialysis? Journal of Renal Care 2022; 49(3): 170-90	Did not fully apply the JBI approach
21. McTague K, Prizeman G, Shelly S, Eustace‐Cook J, McCann E. Youths with asthma and their experiences of self‐management education: a systematic review of qualitative evidence. Journal of Advanced Nursing 2022; 78: 3987-4002	Did not fully apply the JBI approach
22. Ning H, Jiang D, Du Y, Li X, Zhang H, Wu L, *et al.* Older adults’ experiences of implementing exergaming programs: a systematic review and qualitative meta-synthesis. Age and Ageing 2022; 51(12): afac251	Did not fully apply the JBI approach
23. Om P, Whitehead L, Vafeas C, Towell-Barnard A. A qualitative systematic review on the experiences of homelessness among older adults. BMC Geriatrics 2022; 22: 1-10	Did not fully apply the JBI approach
24. Poirier B, Sethi S, Hedges J, Jamieson L. Building an understanding of Indigenous health workers’ role in oral health: a qualitative systematic review. Community Dentistry and Oral Epidemiology 2022; 51(2): 168-79	Did not fully apply the JBI approach
25. Qureshi S, Latif A, Condon L, Akyea RK, Kai J, Qureshi N. Understanding the barriers and enablers of pharmacogenomic testing in primary care: a qualitative systematic review with meta-aggregation synthesis. Pharmacogenomics 2022; 23: 135-54	Did not fully apply the JBI approach
26. Roberts C, Toohey K, Paterson C. The experiences and unmet supportive care needs of partners of men diagnosed with prostate cancer: a meta-aggregation systematic review. Cancer Nurs 2024 [online ahead of print]	Did not fully apply the JBI approach
27. Rogers F, Rashidi A, Ewens B. Education and support for erectile dysfunction and penile rehabilitation post prostatectomy: a qualitative systematic review. International Journal of Nursing Studies 2022; 130: 104212	Did not fully apply the JBI approach
28. See Toh WXS, Lim WHJ, Yobas P, Lim S. The experiences of spousal and adult child caregivers of stroke survivors in transitional care: a qualitative systematic review. Journal of Advanced Nursing 2022; 78: 3897-929	Did not fully apply the JBI approach
29. Shen Y, Zhang C, Valimaki MA, Qian H, Mohammadi L, Chi Y, *et al.* Why do men who have sex with men practice condomless sex? A systematic review and meta-synthesis. BMC Infectious Diseases 2022; 22: 1-19	Did not fully apply the JBI approach
30. Shi Y, Li W, Duan F, Pu S, Peng H, Ha M, *et al.* Factors promoting shared decision-making in renal replacement therapy for patients with end-stage kidney disease: systematic review and qualitative meta-synthesis. International Urology and Nephrology 2022; 54: 553-574	Did not fully apply the JBI approach
31. Simpson R, Simpson S, Wasilewski M, Mercer S, Lawrence M. Mindfulness-based interventions for people with multiple sclerosis: a systematic review and meta-aggregation of qualitative research studies. Disability and Rehabilitation 2022; 44: 6179-93	Did not fully apply the JBI approach
32. Sugiarto A, Lee C-W, Huruta AD. A systematic review of the sustainable campus concept. Behavioral Sciences (Basel, Switzerland) 2022; 12	Was not a qualitative systematic review
33. Tian J, Zhou F, Zhang XG, Wang HY, Peng SH, Li X, *et al.* Experience of physical activity in patients with COPD: a systematic review and qualitative meta-synthesis. Geriatric Nursing 2022; 47: 211-19	Did not fully apply the JBI approach
34. Tringale M, Stephen G, Boylan A-M, Heneghan C. Integrating patient values and preferences in healthcare: a systematic review of qualitative evidence. BMJ Open 2022; 12: e067268	Did not fully apply the JBI approach
35. Wang L, Yao Q, Zhang YP, Xia YL, Gu Y, Zhou HC. [Systematic evaluation of qualitative research on the real experience of burn patients during rehabilitation]. Zhonghua Shao Shang Yu Chuang Mian Xiu Fu Za Zhi. 2022; 38: 69-76 [Chinese]	Did not fully apply the JBI approach
36. Waterfield D, Barnason S. The integration of care ethics and nursing workload: a qualitative systematic review. Journal of Nursing Management 2022; 30: 2194-206	Did not fully apply the JBI approach
37. Yu Z, Shao Q, Hou K, Wang Y and Sun X. The experiences of caregivers of children with epiljpgy: a meta-synthesis of qualitative research studies. Frontiers in Psychiatry 2022; 13: 987892	Did not fully apply the JBI approach
38. Zhang H, Wu Y, Wang N, Sun X, Wang Y, Zhang Y. Caregivers’ experiences and perspectives on caring for the elderly during the COVID‐19 pandemic: a qualitative systematic review. Journal of Nursing Management, 2022; 30: 3972-95	Did not fully apply the JBI approach
39. Zhang J, Zhou F, Jiang J, Duan X, Yang X. effective teaching behaviors of clinical nursing teachers: a qualitative meta-synthesis. Frontiers in Public Health 2022; 10: 883204	Did not fully apply the JBI approach
40. Zheng X, Qian M, Ye X, Zhang M, Zhan C, Li H, *et al.* Implications for long COVID: a systematic review and meta-aggregation of experience of patients diagnosed with COVID-19. Journal of Clinical Nursing 2022; 33(1): 40-57	Did not fully apply the JBI approach

## Appendix III: Full version of data extraction template

**Table:**

Criterion	Elaboration and clarifications
**Citation details**	Reference
**Review aim and objectives – as stated**	State/copy the review aim/objectives
**Review aim and objectives – focus**	• Is the review focused explicitly on an EDI issue? • Yes (Explicit) • No (No mention or inference from the text that social justice/equity is a major focus or concern in this particular review) • Unclear (Implicit, but not directly discussed) • Eg, Is the review explicitly being undertaken in order to address, explore or illuminate inequalities, marginalization or exclusion? • Eg, Does the review aim include a desire or intention to promote social justice/equity as part of its enquiry? • This means that the review needs to have a social justice/EDI orientation (ie. not just [*by chance*] focusing on a population listed under PROGRESS-Plus)
**Review aim and objectives – framing**	• Is the review framed as “generic”/universal issue? or specific to a particular population or context? • Ie, Even where a review has a social justice orientation (eg, seeking to understand the experience of a marginalized group), does it present the group as a generic group (eg, “low income”) or does it address a specific low income population or context? • This question is designed to explore how reviews take context and intersectionality into account
**Geographical focus of review – stated**	• Global/something else?
**Geographical focus – rationale**	• EDI related or not: Yes/No • Is the geographical focus selected and justified in relation to the need to understand/explore EDI and social justice within the phenomenon of interest? • For example, a review may decide to focus on a specific region or group of countries due to having a shared context aiding analysis and transferability, rather than because the geographical focus illuminates a social justice/EDI issue
**Population focus of the review – stated**	• Describe the population(s) considered within the review
**Population focus – rationale**	• EDI related or not: Yes/No • Is the population focus selected and justified in relation to the need to understand/explore EDI and social justice within the phenomenon of interest? • For example, a review may decide to focus on a specific population or sub-population due to having a shared context aiding analysis and transferability, rather than because the specific population illuminates or addresses a social justice/EDI issue
**Number of included studies in the review**	• N =
**Knowledge user involvement – present/absent**	• Are knowledge users involved in the review?
**Knowledge user – contribution and reflection**	• Has knowledge-user involvement been fully described and their contribution made explicit?
**Knowledge user – stages**	• At what stages have knowledge users been involved?
**Reflexivity – review team**	• Is there a description of the review team (beyond simple author affiliation)?
**Reflexivity – descriptive**	• Is there a detailed statement of reflexivity (ie, the team’s standpoint, positionality, roles, reasons for doing the review)?
**Reflexivity – analytical**	• Does the reflexive account include a consideration of how the various standpoints impacted upon the review process? • Eg, What strategies have been employed? • Eg, Discussion about how did the team’s lens/positionality/assumptions shape the review design but also the findings/knowledge claims? • Eg, How were different positionalities navigated and applied during the review process?
**Search – databases**	• Have nontraditional sources been considered (eg, sources from the global South or databases that index non-English-language journals)?
**Search – gray literature**	• Has relevant gray literature been considered?
**Search – language**	• Have non-English-language sources been considered?
**Critical appraisal – reflexivity reporting**	• Has the narrative around critical appraisal described reflexivity within the included papers?
**Critical appraisal – reflexivity reflection**	• Has the narrative around critical appraisal presented a critical evaluation and reflection on the nature of reflexivity within the included papers? • Ie, Has the review team paid attention to how issues of reflexivity may have influenced the quality of their included evidence?
**Critical appraisal – EDI reporting**	• Has the narrative around critical appraisal described any EDI issues within the included papers?
**Critical appraisal – EDI reflection**	• Has the narrative around critical appraisal critically considered and reflected upon EDI issues within the included papers?
**Data extraction**	• Which PROGRESS-Plus characteristics have been extracted (from the included papers)? • Categorize these as far as possible against the PROGRESS-Plus criteria
**Data synthesis – variation**	• Has the analysis explicitly explored or illuminated variations and nuances in experience/outcomes/processes in relation to PROGRESS-Plus characteristics? • Eg, Has there been a subgroup or sensitivity analysis? • Eg, Have outliers been considered and explained (ie, findings that do not easily fit into the main thematic patterns) • This question relates to the extent to which a review team has or has not incorporated an intersectionality lens into its analysis
**Data synthesis – representation**	• Has the issue of power and/or representation been considered within the synthesis? • Eg, Has the synthesis explored differences between groups or contexts and tried to explain these in relation to structural advantages or disadvantages? • Eg, Has the synthesis explored the main findings in relation to issues of inclusion/exclusion/diversity (eg, whose voices are being heard or silenced)?
**Discussion – EDI focus**	• Has the Discussion included a consideration of EDI issues? (may need to categorize) • Eg, Has the review team highlighted the fact that particular groups or regions are under-represented in the evidence? • Eg, Has the review team called for a more social justice or equity oriented approach to future research or action?
**Discussion – transferability**	• Has the Discussion included a consideration of transferability to populations or contexts of disadvantage or underrepresentation?
**Strengths/weaknesses of the review**	• Have strengths or weaknesses related to EDI issues been identified? (may need to categorize)
**Recommendations**	• Have any recommendations related specifically to EDI, health equity or social justice been made?
**Confidence in the review findings**	• Has the ConQual assessment included/or been influenced by any EDI considerations? Yes/No • Eg, Has the team provided a rationale for downgrading a Synthesized Finding due to a lack of reflexivity in the underpinning evidence or due to a lack of credibility of findings due to EDI concerns?

EDI, equity, diversity, and inclusion.

## Appendix IV: Characteristics of included reviews

**Table:**

Authors	Review aim and objectives	Geographical focus of review	No. of included studies in the review
Abdul Rahman *et al.* 2022^55^	To review qualitative studies on the lived experience of individuals diagnosed with leprosy, the impact of the disease, and how they coped with the disease burden	Global	49
Bayo *et al.* 2022^56^	To explore the experiences of mothers with the practice of kangaroo mother care for preterm neonates at home in sub-Saharan Africa	Sub-Saharan Africa	6
Casaleiro *et al.* 2022^57^	To identify the spiritual aspects of the family caregivers’ experiences when caring for a community-dwelling adult with severe mental illness	Global	26
Chang *et al.* 2022^58^	To explore women’s, peer supporters’ and healthcare professionals’ views and experiences of breastfeeding peer support	Global	22
Conti *et al.* 2022^59^	To identify self-care behaviors, skills, and strategies performed by individuals with spinal cord injury	Global	12
Cooper-Stanton *et al.* 2022^60^	To explore on the experiences of men with chronic lymphoedema. This qualitative systematic literature review aims to address an imbalance in the current evidence base between men and women diagnosed with lymphoedema	Global	22
Cramm *et al.* 2022^61^	To describe the experiences of children growing up in military families with a parent who has military-related post-traumatic stress disorder	United States, Canada, and Australia	12
Davenport *et al.* 2022^62^	To understand fathers’ experiences of depression in the perinatal period, including how they recognize their depression, the emotions they experience, the impact of depression on their relationships, and their help-seeking behaviors and support	Organisation for Economic Co-operation and Development (OECD) countries	9
Dymmott *et al.* 2022^63^	To explore experiences of early career rural and remote allied health professionals and doctors to better understand both the profession specific and common factors that influence their experience	High-income countries	30
Egan *et al.* 2022^64^	To synthesize studies that investigated the lived experience of barriers and facilitators to educational access and excellence for students with disabilities in low- and middle-income African countries	Low- and middle-income countries	13
Emmett *et al.* 2022^65^	To identify and explore the experiences of health professionals towards using mobile electrocardiogram (ECG) technology	Global	6
Fu *et al.* 2022^66^	To explore the experiences of breast cancer survivors with lymphedema self-management	Global	24
Hallam *et al.* 2022^67^	To explore care home staff’s perceptions of physical activity among older adults	Global	25
Hassanein *et al.* 2022^68^	To explore experiences and views of parents, children, and professionals on the prevention of second-hand smoke exposure to women and children in Middle Eastern countries	Middle Eastern countries	3
Jay *et al.* 2022^69^	To explore experiences of recovery among adults with a mental illness using visual art methods	Global	14
Kabiri *et al.* 2022^70^	To summarize policies for the prevention of common gastrointestinal cancers worldwide	Global	9
Kassam *et al.* 2022^71^	To explore experiences of nurses providing care within various health care delivery environments to involuntary migrant women who are experiencing pregnancy, birth, or post-birth. Studies included in this review characterized involuntary migrants predominantly as having varying migrant statuses	Global	23
Koto *et al.* 2022^72^	To investigate the experiences of patients with lysosomal storage disorders who are receiving enzyme-replacement therapy and the experiences of their family members	Global	7
Li *et al.* 2022^73^	To identify and synthesize the available evidence of bowel symptom experiences of patients with rectal cancer after sphincter-preserving surgery (SPS)	Global	7
Lim *et al.* 2022^74^	To synthesise the best available evidence exploring nurses’ experiences in managing delirium of older persons in acute care wards	Global	31
Maehara *et al.* 2022^75^	To explore experiences of the transition to motherhood among pregnant women following assisted reproductive technology	Global	7
Matarese *et al.* 2022^76^	To explore the experiences of health care personnel with promoting a sense of home for older adults living in residential care facilities	Global	7
May *et al.* 2022^77^	To examine the perceptions and experiences of family members of emergency first responders (EFRs) with post-traumatic stress disorder (PTSD)	Global	5
McCloskey *et al.* 2022^78^	To explore the experiences of faculty and staff nurses working with nursing students in clinical placement in residential aged care facilities	Global	6
Meng *et al.* 2022^79^	To explore advanced cancer patients’ experiences with low socioeconomic status, and then to help provide targeted and effective strategies to improve their quality of life	Global	9
Min *et al.* 2022^80^	To understand the experiences of children, young adults and their carers living with juvenile idiopathic arthritis in any setting	Global	10
Morbach *et al.* 2022^81^	To synthesize evidence related to aspects of the development of older adults in qualitative studies that have analytical psychology as a reference	Global	5
Mostafaei *et al.* 2022^82^	To synthesize patients’ and providers’ experiences while using telemedicine in cancer care during the COVID-19 pandemic	Global	19
Nan *et al.* 2022^83^	To explore the perspective of older adults, caregivers, and healthcare providers on frailty screening and determine the enablers and barriers to implementing frailty screening in primary care	Global	6
O’Shea *et al.* 2022^84^	To identify barriers and facilitators related to self-management from the perspectives of people with shoulder pain and clinicians	Global	20
Parsons *et al.* 2022^85^	To synthesize the qualitative literature on the experience of upwards violence in nursing workplaces directed towards nurse leaders who have authority over those who direct the violence towards them	Global	6
Qiu *et al.* 2022^86^	To identify the barriers and enablers that affect physical activity participation in people with venous leg ulcers	Global	18
Small 1 *et al.* 2022^87^	To explore barriers in work disability policies with respect to labor market engagement and to explore facilitators in work disability policies with respect to labor market engagement	Global	44
Small 2 *et al.* 2022^88^	To identify experiences of women who smoked tobacco during pregnancy or postnatally (or both) concerning health care providers’ interactions with them about their smoking, when such interactions occurred during contact for prenatal or postnatal health care in any health care setting; and to synthesize the research findings for recommendations to strengthen health care providers’ interventions regarding smoking during pregnancy and smoking during the postnatal period	Global	57
Suh *et al.* 2022^89^	To provide a comprehensive understanding of the challenges experienced by children with Tourette’s syndrome (TS) and their caregivers with the aim of providing more effective treatment and services for them	Global	8
Tanywe *et al.* 2022^90^	To synthesize the perceptions and practices of community members relating to trachoma in Africa	Africa	7
Taylor *et al.* 2022^91^	To synthesize the literature regarding the experiences of new graduate nurses working in a pediatric setting	Global	9
Tuomikoski *et al.* 2022^92^	To explore experiences of people with progressive memory disorders who are involved in non-pharmacological interventions	Global	46
Whitehead *et al.* 2022^93^	To explore nurses’ perceptions and beliefs related to the care of adults living with multimorbidity	Global	11
Yao Bian *et al.* 2022^94^	To integrate the psychological experience of infected individuals during the pandemic	Global	7
Zheng *et al.* 2022^95^	To explore the experiences, perspectives, and consequences of pregnant women experiencing motherhood during the COVID-19 pandemic	Global	24
Zhu 1 *et al.* 2022^96^	To explore experiences of caring for advanced cancer patients	Global	26
Zhu 2 *et al.* 2022^97^	To synthesize qualitative evidence on nursing students’ experiences with service learning	Global	42
